# Supplementary material for: Potent neutralization by antibodies targeting the MPXV A28 protein
Source: Nat Commun. 2025 Dec 10;16:11455. doi: 10.1038/s41467-025-66344-0 (PMC12748864; doi:10.1038/s41467-025-66344-0)
Supplement: Supplementary file 1 — Supplementary Information [file 41467_2025_66344_MOESM1_ESM.pdf]

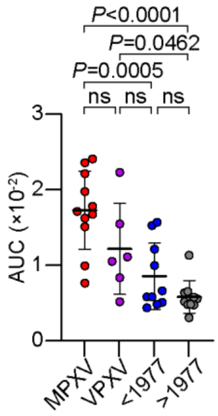

Supplementary Figure 1: Antibody binding to A28. Sera binding to MPXV A28 antigen as measured by ELISA. AUC values of 4 consecutive 4-fold dilutions, starting from 1:100. Mpox convalescent donors, MPXV, are in red (n=11), recently vaccinated donors, VPXV, are in purple (n=6), historic vaccinated donors born before 1977, <1977, are in blue (n=10) and naïve donors born after 1977, >1977, are in gray (n=10). Statistical analysis was performed using One-Way ANOVA with Tukey's multiple comparison correction. ns= non significant. Data are representative of two independent experiments with similar results. Source data are provided as a Source Data file.

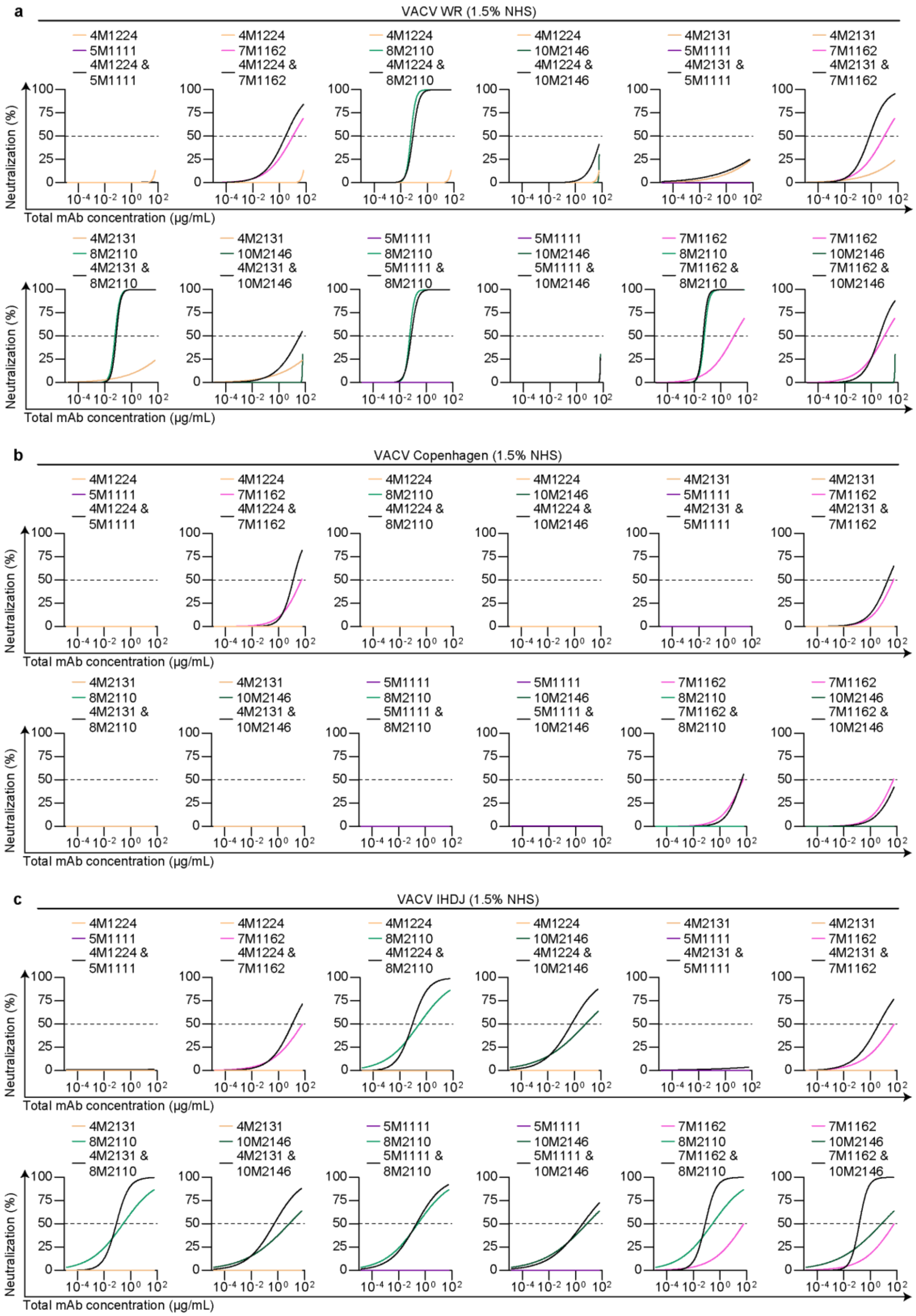
a
VACV WR (1.5% NHS)
4M1224
5M1111
4M1224 & 5M1111
4M1224
7M1162
4M1224 & 7M1162
4M1224
8M2110
4M1224 & 8M2110
4M1224
10M2146
4M1224 & 10M2146
4M2131
5M1111
4M2131 & 5M1111
4M2131
7M1162
4M2131 & 7M1162
4M2131
8M2110
4M2131 & 8M2110
4M2131
10M2146
4M2131 & 10M2146
5M1111
8M2110
5M1111 & 8M2110
5M1111
10M2146
5M1111 & 10M2146
7M1162
8M2110
7M1162 & 8M2110
7M1162
10M2146
7M1162 & 10M2146
Neutralization (%)
Total mAb concentration (μg/mL)
b
VACV Copenhagen (1.5% NHS)
c
VACV IHDJ (1.5% NHS)

Supplementary Figure 2: Increased potency of anti-MPXV mAb combinations. a Neutralization of VACV WR strain by anti-MPXV pairs in the presence of 1.5% NHS (3% during pre-incubation). Neutralization curves of 12 consecutive 4-fold dilutions, starting from 62.5 µg/mL. Each combination, as well as the individual mAbs, is depicted in a different panel. **b** Same as **(a)** but for VACV Copenhagen. **c** Same as **(a)** but for VACV IHDJ. Anti-A35, anti-H3 and anti-A28 mAbs are shown in red, purple and green, respectively. Combinations are in black. Neutralization curves were determined by fitting values using the Agonist vs. normalized response (Variable slopes) nonlinear regression. The percentages of complement sources indicated represent the final concentrations in the infected wells. Source data are provided as a Source Data file.

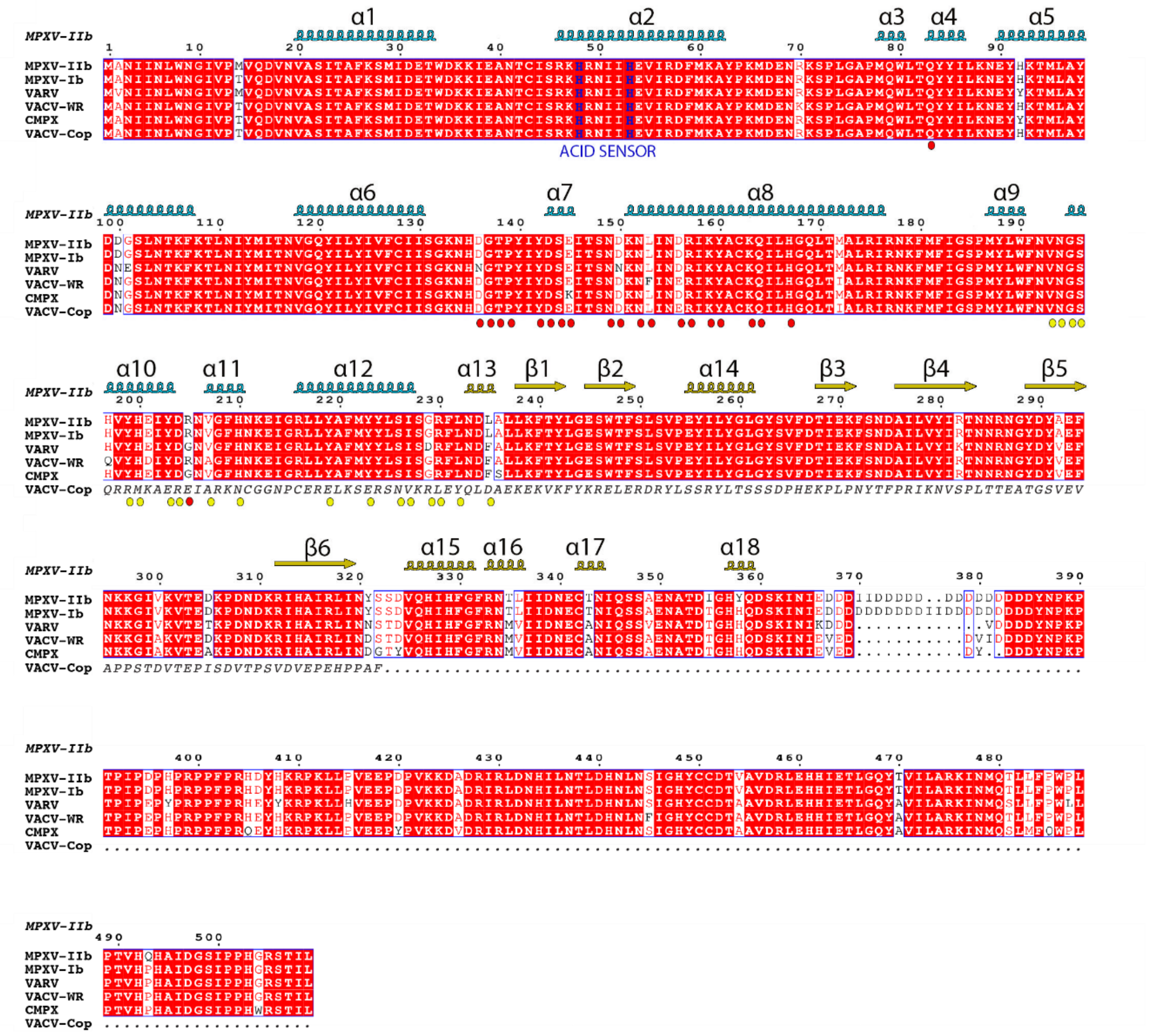

Supplementary Figure 3: Multiple sequence alignment of A28 homologs. A28 homologs (OPG153) from six representatives Orthopoxviruses—MPXV virus sub-clade IIb (MPXV; accession A0A7H0DNC4), MPXV virus sub-clade Ib (Ref-SKU: 012V-06039) Variola virus (VARV; Q89489), Vaccinia virus WR (VACV; P24758), Vaccinia virus Copenhagen (VACV; P21114.1), and Camelpox virus (CMPX; Q8QQ29). Secondary structure elements are labeled above the sequences and colored by domain (NTD in blue, CTD in tan). Strictly conserved residues are highlighted with a red background. Epitope residues (BSA > 10 Å^2) are marked by color-coded spheres below the sequences: red for 10M2146 and yellow for 8M2110.

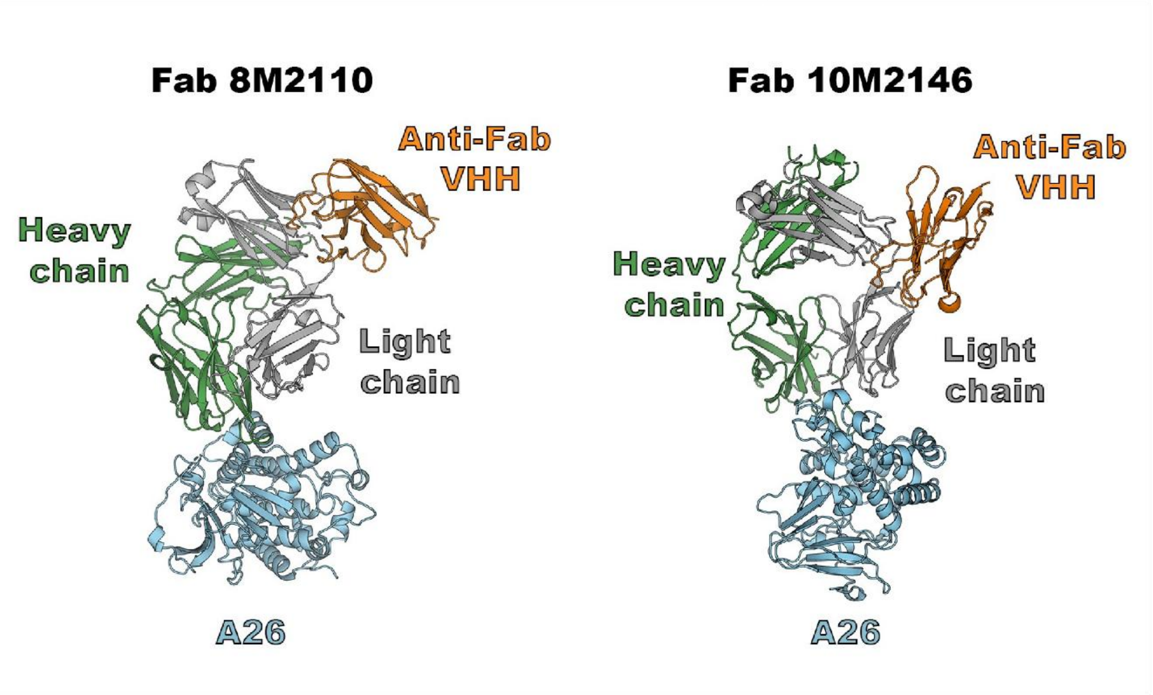

Supplementary Figure 4. Crystal structures of the complexes A26/Fab/anti-Fab VHH. Models of VACV A26/Fab-8M2110/anti-Fab VHH (left panel) and VACV A26/Fab-10M2146/anti-Fab VHH (right panel) complexes depicted in cartoon. A26 is colored cyan, the heavy and light chains of the Fab are colored green and gray, respectively, and the anti-Fab VHH in showed in orange, as indicated.

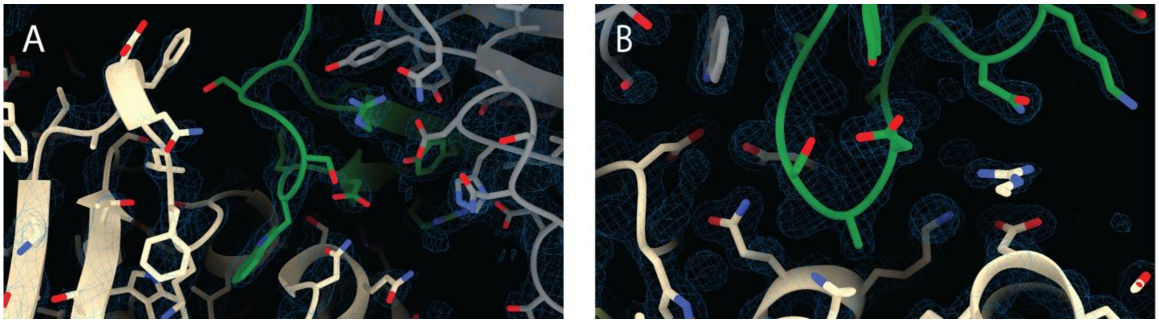

Supplementary Figure 5: Electron density map of the reported crystal structures. a 2Fo–Fc electron density map and model of the CDR3 region in the A26–8M2110 complex. The electron density is shown as a blue mesh at 1.5 σ contour level. A26 is colored pale brown, the 8M2110 heavy chain in green, and the light chain in gray. **b** Electron density map and model of the CDR3 region in the A26–10M2146 complex, represented as in panel **(a)**.

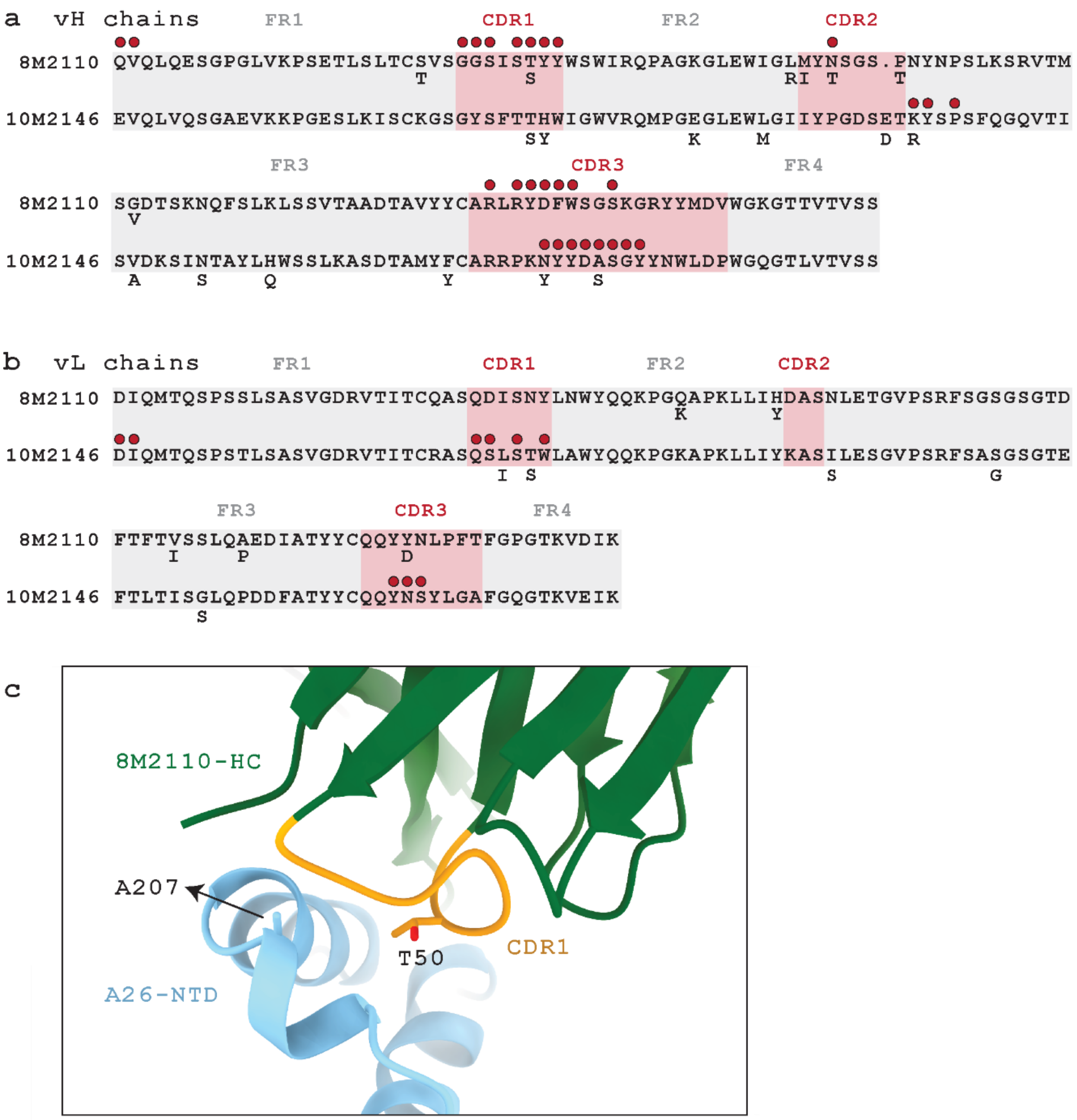

Supplementary Figure 6: Sequence analysis of anti-A28 antibodies. a Sequence alignment of the heavy chains of 8M2110 and 10M2146 with the CDR regions in a red background. The germline residues are indicated underneath. Paratope residues (BSA > 10 Å^2) are marked by red spheres above the sequences. **b** Sequence alignment of the light chains of 8M2110 and 10M2146 annotated as in **(a)**. **c** Close-up view of the A26/8M2110 interacting region around CDR H1 showing the localization of the A207 (V207 in A28) and T50 (S50 in the germline sequence).

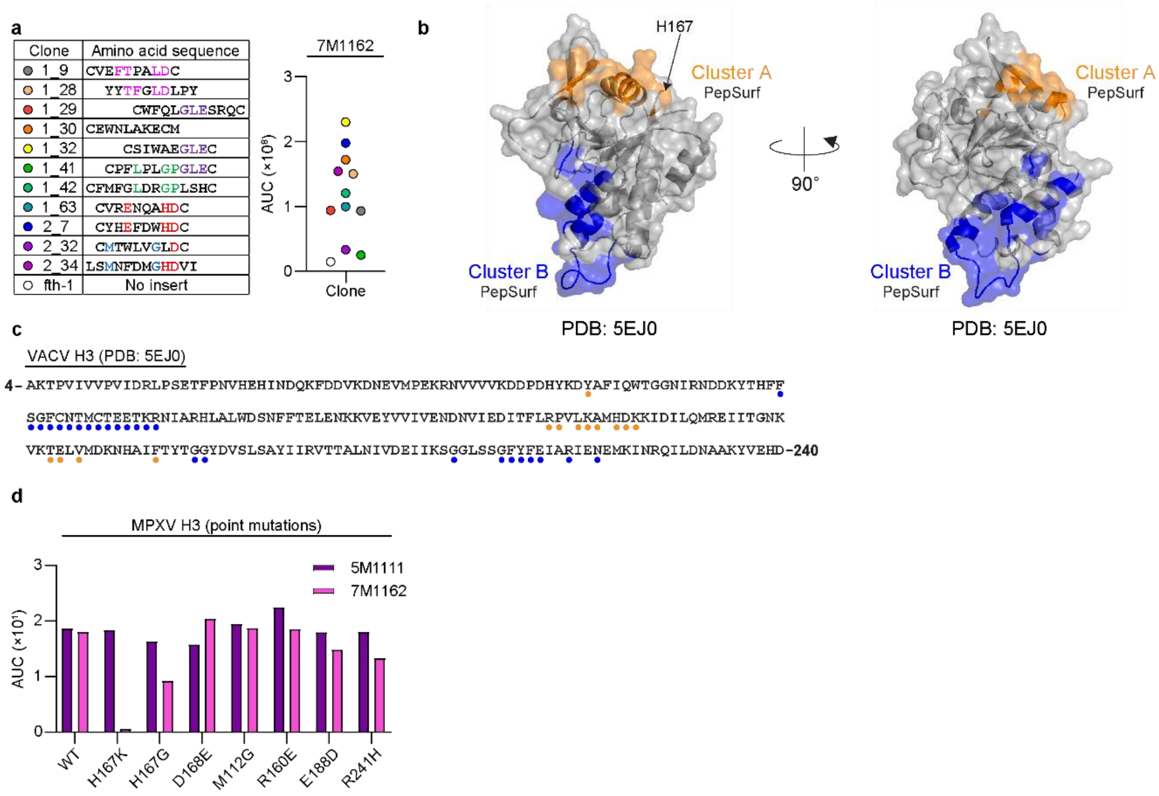

Supplementary Figure 7: Prediction of the epitope of anti-H3 mAb 7M1162. a Left panel: peptide sequences isolated using Biopanning from a random phage display peptide library screened with the anti-H3 mAb 7M1162. Shared motifs are marked by matching colors. Right panel: The AUC values in ELISA of 8 consecutive 4-fold phage dilutions, starting from 4×10^{8} phages per well. fth-1 (a phage without any recombinant peptide) serves as a negative control. **b** MAb 7M1162 predicted epitope on the surface of VACV H3 (PDB: 5EJ0) as predicted by Pepsurf software that aligns the isolated peptides onto the 3D surface of VACV H3. Predicted epitope clusters A and B are in orange and blue, respectively. **c** Sequence representation of predicted epitopes. Inferred epitope residues are marked by color-coded spheres below the sequences: orange for cluster A and blue for cluster B. **d** Binding of 7M1162 (pink) and 5M1111 (purple) to MPXV H3 point mutations as measured by ELISA. AUC values of 6 consecutive 10-fold dilutions, starting from 10

μg/mL. Data are representative of two independent experiments with similar results **(a,d)**.

Source data are provided as a Source Data file.

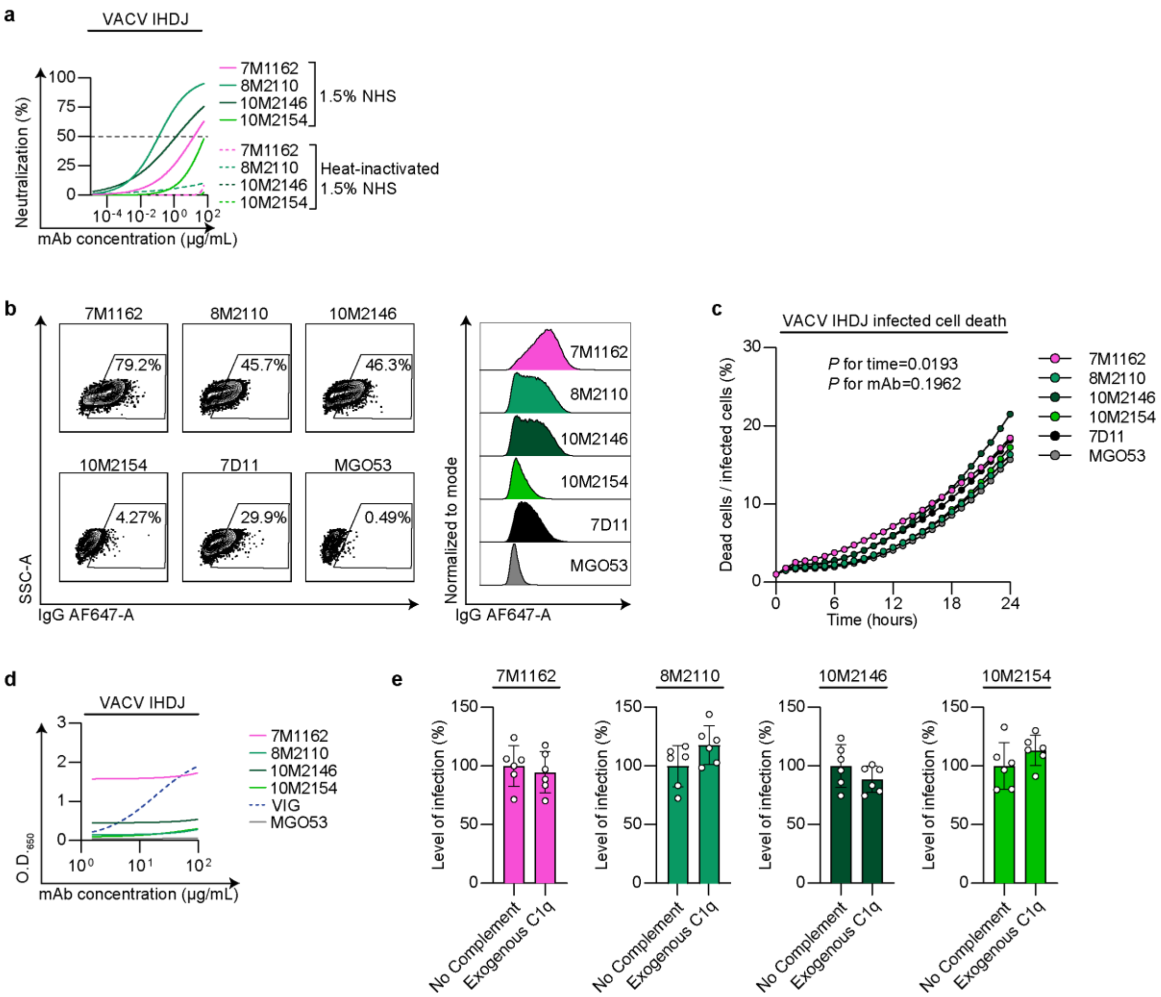

Supplementary Figure 8: Anti-MPXV mAbs do not elicit CDC of VACV-infected cells.

a Neutralization of VACV IHDJ by anti-MPXV mAbs in the presence of 1.5% NHS (3% during pre-incubation; solid lines) versus heat-inactivated NHS (dashed lines). Neutralization curves of 12 consecutive 4-fold dilutions starting from 62.5 μg/mL. Horizontal black dashed line represents 50% neutralization. Complement percentages indicate final concentrations in infected wells. **b** Binding of anti-MPXV mAbs (10 μg/mL) to VACV-infected cells. Left panel: flow cytometry plots; right panel: median fluorescence intensity. **c** Induction of CDC on VACV-infected cells. Baseline cell death was measured at time 0, following addition of 10 μg/mL mAbs in 1.5% NHS, and monitored over 24 hours using a cell death marker and live-cell imaging. **d** Binding curves of anti-MPXV mAbs to

β-propiolactone-inactivated VACV IHDJ by ELISA of 4 consecutive 4-fold dilutions starting from 100 µg/mL. **e** Level of VACV IHDJ infection in the presence of anti-MPXV mAbs at IC_{50} concentrations, with or without 1.2 µg/mL exogenous C1q (equivalent to levels in 1.5% NHS). Anti-H3 and anti-A28 mAbs are shown in magenta and green; anti-M1 (7D11) in black; VIG in dashed blue; MGO53 as an isotype control in gray. IC_{50} concentrations for panel (**e**) were: 15 µg/mL (7M1162), 0.125 µg/mL (8M2110), 1.25 µg/mL (10M2146), and 62.5 µg/mL (10M2154). Statistical analysis for (**c)** was performed using Two-Way repeated measures ANOVA with Dunnett's multiple comparison correction to the MGO53 treated wells as control. Neutralization curves were determined by fitting values using Agonist vs. normalized response (Variable slopes) nonlinear regression for **(a)**. Binding curves were determined by fitting values using the Agonist vs response (three parameters) nonlinear regression for **(b),** Standard deviation of mean are shown in **(e)**. Data are representative of two independent experiments with similar results **(a,b,d,e)**. Source data are provided as a Source Data file.

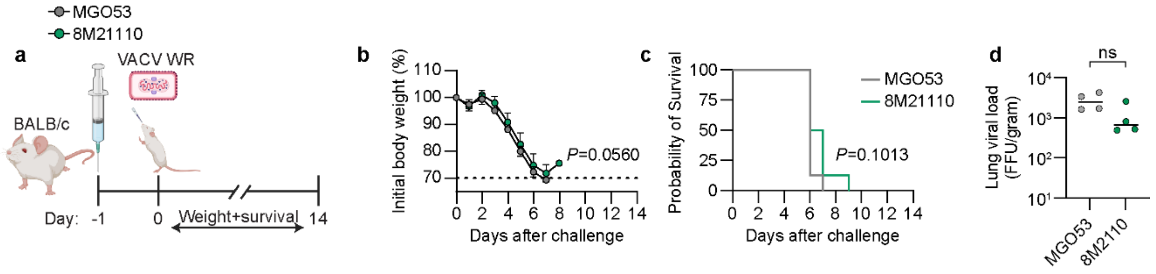

Supplementary Figure 9: Prophylactic administration of A28 mAbs modestly delays the onset of severe disease. a Schematic representation of mice prophylactic mAb transfer and challenge. Female BALB/c mice (n = 8 per group) were immunized I.P. with 200 µg of mAbs 8M2110 (anti-A28) or MGO53 (isotype control). The following day, mice were inoculated I.N. with a lethal dose of VACV WR (2×10^5 PFU) and their weight and survival were monitored for 14 days. Mice that lost more than 30% of their initial weight (or more than 25% and exhibited a core body temperature below 34°C) were considered to have reached the no recovery threshold and were subsequently sacrificed. **b** Body weight changes of initial weight for 8M2110 or MGO53 immunized mice. **c** Kaplan-Meier survival curve of 8M2110 or MGO53 immunized mice. **d** Lung viral load on day 5 post-VACV challenge in BALB/c 8M2110 or MGO53 immunized mice (n = 4 per group). Mice immunized with mAb 8M2110 are represented in green, while those immunized with MGO53 are shown in gray. Body weight comparison analysis was performed using Main-effects analysis for **(b)**. Statistical analysis was conducted by comparing survival curves to the isotype control using the Log-rank (Mantel-Cox) test in **(c)** and by Unpaired T test in **(d)**. ns= non significant. Images were created using *BioRender. Freund, N. (2025)* *https://BioRender.com/y6l0xzo*. Source data are provided as a Source Data file.

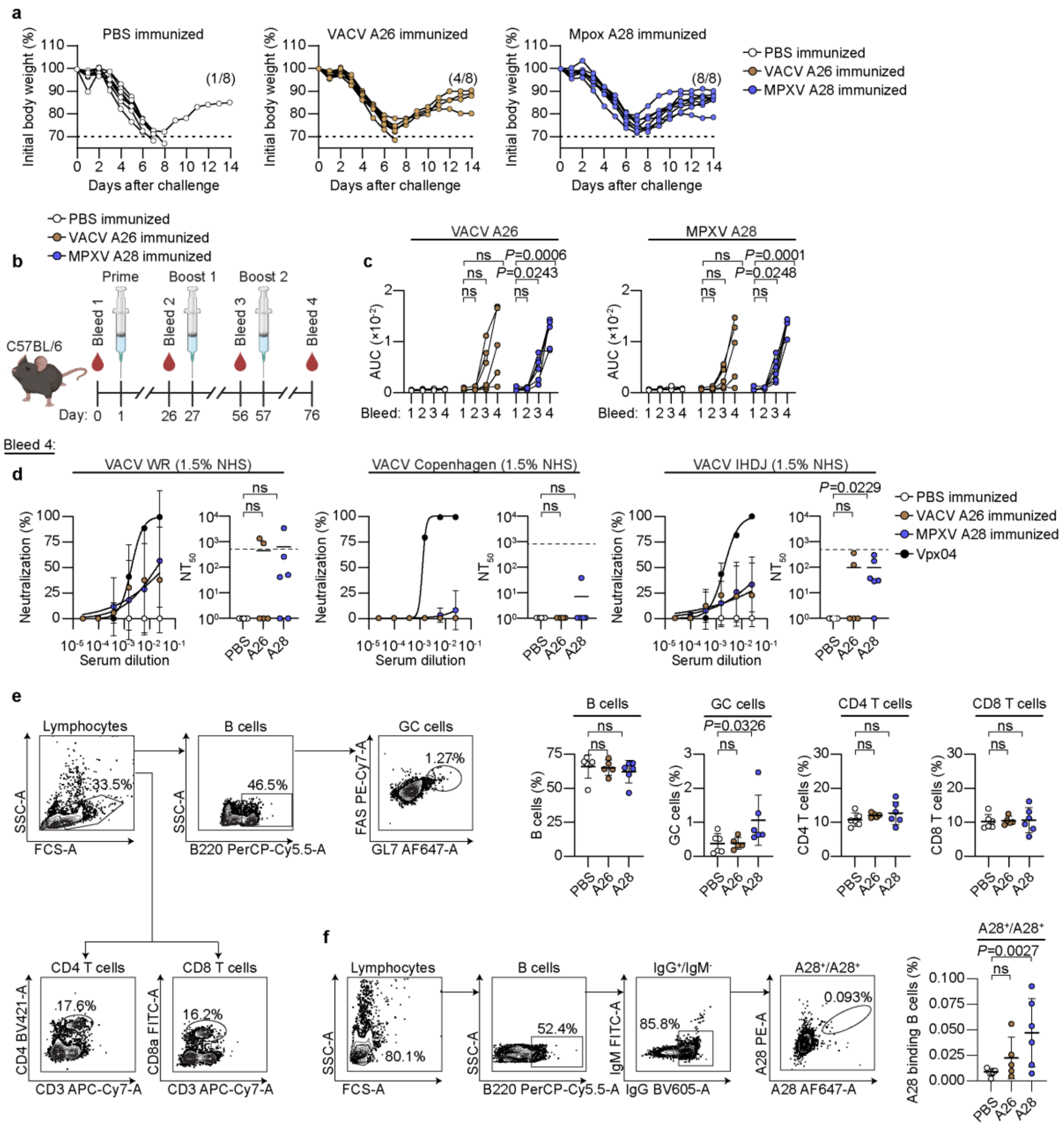

Supplementary Figure 10: A28 vaccination elicits specific antibody and B cell responses. a Body weight changes of individual mice from **Fig. 6a-b**. Numbers in parentheses indicate mice reaching the experimental endpoint. Left panel: PBS-immunized mice. Middle panel: VACV A26–immunized mice. Right panel: MPXV A28–immunized mice. **b** Schematic of the immunization protocol. Female C57BL/6 mice (n = 6 for PBS and A28 groups; n = 7 for A26 group) were immunized I.P. with PBS, MPXV A28, or VACV A26 (10 μg/dose) formulated with alum, following a prime plus two boosts at 3–

4-week intervals. Blood was collected before each inoculation, sera heat-inactivated, and mice sacrificed three weeks after the final boost for blood and spleen collection. **c** Time course analysis of sera binding as measured by ELISA. AUC values of 4 consecutive 4-fold dilutions starting at 1:100. Left panel: VACV A26 antigen. Right panel: MPXV A28 antigen. **d** Neutralization of VACV WR, Copenhagen, and IHDJ strains by bleed-4 sera in the presence of 1.5% NHS (3% during pre-incubation). NT_{50} values calculated from six serial 4-fold dilutions starting at 1:40. **e** Flow cytometry analysis of splenocyte populations. Left panel: representative gating from an A28-vaccinated mouse. Right panels: percentages of total B cells, GC B cells, CD4 T cells, and CD8 T cells. **f** Flow cytometry of MPXV A28–binding B cells from vaccinated mouse spleens. Left panel: representative gating from an A28-vaccinated mouse. Right panel: percentage of A28-binding B cells. PBS-immunized mice are in white (n=6), VACV A26–immunized in brown (n=5), and MPXV A28–immunized in blue (n=6). Sera from a triple-vaccinated human (Vpx04) is in black. Statistical analyses: for (**b)**, two-way repeated measures ANOVA with Dunnett's correction versus baseline (bleed 1); for (**c–d)**, Kruskal-Wallis with Dunn's correction; for (**e)**, One-Way ANOVA with Dunnett's correction. The percentages of complement sources indicated represent the final concentrations in the infected wells. Images were created using *BioRender. Freund, N. (2025)* *https://BioRender.com/y6l0xzo*. ns= non significant. Data are representative of two independent experiments with similar results **(c,d)**. Source data are provided as a Source Data file.

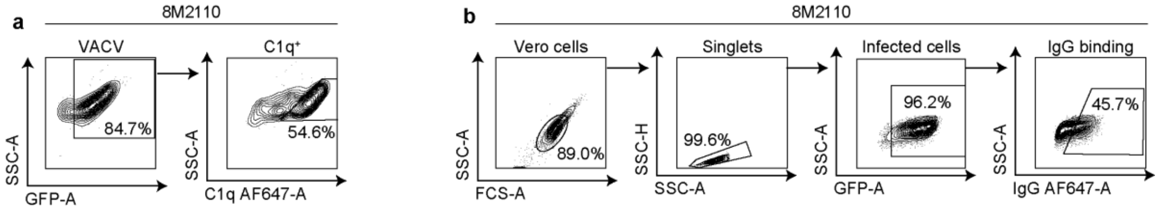

Supplementary Figure 11: Full gating strategy for flow cytometry assays. a. Gating strategy corresponding to **(Fig. 5e)**. Purified VACV IHDJ virions (~5×10^6 PFU) were incubated with 1.5% NHS and 10 µg/mL mAbs. Samples were analyzed by flow cytometry, with virions gated based on GFP signal to identify virion-positive events and assessed for C1q deposition using anti-C1q-AF647 staining. Representative plot for anti-A28 mAb, 8M2110, is presented. **b** Gating strategy corresponding to **(Supplementary Fig. 8b**). VACV IHDJ-infected cells were incubated with 10 µg/mL of anti-MPXV mAbs and analyzed by flow cytometry. Cells were gated based on size, granularity, and GFP signal to identify infected populations, and IgG binding was measured using anti-human IgG-AF647. Representative plot for anti-A28 mAb, 8M2110, is presented.

Supplementary table 1: Study participants clinical data

Patient ID	Birth year	Gender	Infection	Time from infection to time point 1 (V1) sample collection (days)	Time from infection to time point 2 (V2) sample collection (days)
Mpx04	1985	Male	Mpox	48	311
Mpx05	1983	Male	Mpox	49	N/A
Mpx07	1996	Male	Mpox	47	N/A
Mpx08	1986	Male	Mpox	50[a]	322[a]
Mpx09	1999	Male	Mpox	51	322
Mpx10	1999	Male	Mpox	51	322
Mpx11	1989	Male	Mpox	51[a]	308[a]

[a] Date of infection not known- set as 2 weeks before diagnosis.

N/A- not applicable

Supplementary table 2. Crystallographic Statistics		
	**A26 + Fab 8M2110 + antiFab V_HH**	**A26 + Fab 10M2146 + antiFab V_HH**
**Data collection**		
Space group	P 32 2 1	C 2 2 21
Unit cell parameters		
a (Å)	119.4	122.4
b (Å)	119.4	157.8
c (Å)	632.8	141.4
α (º)	90	90
β (º)	90	90
φ (º)	120	90
Resolution (Å)	39.09 – 2.90	39.93 – 1.90
Last resolution bin (Å)[a]	2.95 – 2.90	1.93 – 1.90
Total observations	2124826 (105841)	1382286 (68092)
Unique reflections	117931 (5717)	107567 (5260)
Completeness (%)[a]	100 (100)	100 (100)
Redundancy [a]	18.0 (18.5)	12.9 (12.9)
<I/s> [a]	8.6 (2.1)	10.2 (1.8)
R_{sym} (%)[a,b]	34.5 (167.0)	16.1 (139.0)
$CC_{1/2}$	0.987 (0.638)	0.997 (0.680)
Resolution I/s > 2	2.90	1.93
Wilson B factor (Å^2)	41.9	18.6
**Refinement**		
PDB accesion code	pdb_00009QT3	pdb_00009SHD
Resolution (Å)	39.08 – 2.90	39.93 – 1.90
Last resolution bin (Å)[a]	2.93 – 2.90	1.92 – 1.90
Number of reflections	223523 (6995)	107508 (5432)
Number of Rfree reflections	11388 (340)	3397 (183)
B refinement	ISOTROPIC + TLS	
Number of TLS groups	16	4
Rfactor (%) [a,c]	20.1 (30.7)	15.9 (27.3)
Rfree (%) [a,c]	23.5 (35.6)	19.3 (33.2)
Mean B value (Å^2)		
Protein	43.1	26.0
Waters	30.5	37.5
Root mean square deviations		
Bond lengths (Å)	0.002	0.009
Bond angles (º)	0.503	0.958
Ramachandran favored/outliers (%)	97.3/0.06	97.8/0.00

[a] Data for the last resolution shell are in parenthesis

[b] Rsym = Σ|Ii - <Ii>|/ Σ Ii, where Ii is the observed intensity and <Ii> is the average intensity obtained from multiple observations of symmetry-related reflections.

[c] Rwork = Σ‖Fobs(hkl)|-|Fcalc(hkl)‖/ Σ|Fobs(hkl)|
