# Supplementary material for: Potent neutralization by antibodies targeting the MPXV A28 protein
Source: Nat Commun. 2025 Dec 10;16:11455. doi: 10.1038/s41467-025-66344-0 (PMC12748864; doi:10.1038/s41467-025-66344-0)
Supplement: Supplementary file 2 — Description of Additional Supplementary Files [file 41467_2025_66344_MOESM2_ESM.pdf]

Description of additional supplementary files

Title: Supplementary data 1: Anti-MPXV mAbs sequence data.

Description: V(D)J sequence features of monoclonal antibodies isolated from convalescent donors following Mpox virus infection. The table summarizes, for each antibody, the donor of origin, sampling time point, and recognized viral antigen. Shown are the Heavy and Light chain variable gene usage (V, D, and J segments), antibody isotype, complementarity-determining region 3 (CDR3) amino acid sequence and length, as well as the percentage of somatic mutations relative to the corresponding germline gene segments.
