# Supplementary material for: Potent neutralization by antibodies targeting the MPXV A28 protein
Source: Nat Commun. 2025 Dec 10;16:11455. doi: 10.1038/s41467-025-66344-0 (PMC12748864; doi:10.1038/s41467-025-66344-0)
Supplement: Supplementary file 4 — Reporting Summary [file 41467_2025_66344_MOESM4_ESM.pdf]

nature portfolio

Corresponding author(s): Natalia T Freund

Last updated by author(s): Oct 13, 2025

Reporting Summary

Nature Portfolio wishes to improve the reproducibility of the work that we publish. This form provides structure for consistency and transparency in reporting. For further information on Nature Portfolio policies, see our Editorial Policies and the Editorial Policy Checklist.

Statistics

For all statistical analyses, confirm that the following items are present in the figure legend, table legend, main text, or Methods section.

n/a	Confirmed	
☐	☒	The exact sample size (*n*) for each experimental group/condition, given as a discrete number and unit of measurement
☒	☐	A statement on whether measurements were taken from distinct samples or whether the same sample was measured repeatedly
☐	☒	The statistical test(s) used AND whether they are one- or two-sided<br>*Only common tests should be described solely by name; describe more complex techniques in the Methods section.*
☒	☐	A description of all covariates tested
☐	☒	A description of any assumptions or corrections, such as tests of normality and adjustment for multiple comparisons
☐	☒	A full description of the statistical parameters including central tendency (e.g. means) or other basic estimates (e.g. regression coefficient) AND variation (e.g. standard deviation) or associated estimates of uncertainty (e.g. confidence intervals)
☐	☒	For null hypothesis testing, the test statistic (e.g. *F*, *t*, *r*) with confidence intervals, effect sizes, degrees of freedom and *P* value noted<br>*Give P values as exact values whenever suitable.*
☒	☐	For Bayesian analysis, information on the choice of priors and Markov chain Monte Carlo settings
☒	☐	For hierarchical and complex designs, identification of the appropriate level for tests and full reporting of outcomes
☒	☐	Estimates of effect sizes (e.g. Cohen's *d*, Pearson's *r*), indicating how they were calculated

Our web collection on statistics for biologists contains articles on many of the points above.

Software and code

Policy information about availability of computer code

Data collection	Diffraction data of PDB 9SHD and 9QT3 were collected using SOLEIL synchrotron and were processed using XDS (version January 10, 2022) initial phases were obtained using PHASER software.<br>Structural models were built and refined iteratively using phenix.refine (Phenix version 1.19.2-4158) and Coot.<br>Model validation was performed with MolProbity.<br>Fluorescent cell images were analyzed using the IncuCyte live-cell analysis system for VACV and Harmony software for Mpox.<br>Protein 3D structures were visualized using Pymol software versionv 3.0.3 or 3.1.3.1.<br>Western blot images were acquired in Chemidoc imaging system (Bio-Rad).<br>TEM images were colected using a JEM-1400 transmission electron microscope (JEOL Ltd, Tokyo, Japan) operating at 80 kV.<br>Flow Cytometry data collection was performed using the CytoDLEX 4L (Beckman Coulter)
Data analysis	Statistical analysis was carried out using the GraphPad Prism software version 9.4.1.<br>Analysis of Flow Cytometry data was carried out using FlowJo software version 10.8.<br>Fluorescent cell images were analyzed using the IncuCyte live-cell analysis system for VACV and Harmony software for Mpox.<br>Protein 3D structures were visualized using Pymol software version 3.1.3.1.<br>IgH/IgL sequences were analyzed and annotated using IgBLAST and the IMGT database for sequence quality, viability, Gamma chain subtype (IgH only), VHDHJH or VLJL gene usage, mutational load, and CDR3 length.<br>Diffraction data of structural studies were processed using XDS (version January 10, 2022), and initial phases were obtained using PHASER software.<br>Structural models were built and refined iteratively using phenix.refine (Phenix version 1.19.2-4158) and Coot, applying isotropic B-factor and one TLS group per chain. Model validation was performed with MolProbity.

Unique peptide sequences were used for epitope prediction based on the VACV H3 structure PDB:5EJ0 using the PepSurf software.

For manuscripts utilizing custom algorithms or software that are central to the research but not yet described in published literature, software must be made available to editors and reviewers. We strongly encourage code deposition in a community repository (e.g. GitHub). See the Nature Portfolio guidelines for submitting code & software for further information.

Data

Policy information about availability of data

All manuscripts must include a data availability statement. This statement should provide the following information, where applicable:

- Accession codes, unique identifiers, or web links for publicly available datasets
- A description of any restrictions on data availability
- For clinical datasets or third party data, please ensure that the statement adheres to our policy

Data and materials availability:
The atomic coordinates of mAbs 10M2146 and 8M2110 with the VACV A26 will be deposited in the PDB as 9SHD and 9QT3.
Further information and requests for resources and reagents should be directed to and will be fulfilled by the lead contact, Natalia T Freund (nfreund@tauex.tau.ac.il).

Research involving human participants, their data, or biological material

Policy information about studies with human participants or human data. See also policy information about sex, gender (identity/presentation), and sexual orientation and race, ethnicity and racism.

Reporting on sex and gender	The study included only male participants, as Mpox clade 2b infections in Israel during the 2022 outbreak disproportionately affected males. Sex was considered in the study design, and inclusion criteria reflected the epidemiology of the outbreak. Participant sex was determined from medical records at the time of diagnosis. Gender identity was not separately assessed, as it was not relevant to the research question.Because all participants were male, disaggregation of results by sex or gender was not applicable. No sex- or gender-based comparative analyses were performed for human data.
Reporting on race, ethnicity, or other socially relevant groupings	Race, ethnicity, or other socially relevant groupings were not considered in this study design.
Population characteristics	All study participants were male above 18 years of age and diagnosed with Mpox prior to data collection.
Recruitment	Study participant were recruited after their diagnosis of Mpox in Israel between the months of May-June 2022.
Ethics oversight	The Tel Aviv University Institutional Review Board (IRB) approved all studies involving patient enrollment, sample collection, and clinical follow-up (protocol number 0005243–1; Helsinki approval number 0384-22-TLV)

Note that full information on the approval of the study protocol must also be provided in the manuscript.

Field-specific reporting

Please select the one below that is the best fit for your research. If you are not sure, read the appropriate sections before making your selection.

[x] Life sciences   [ ] Behavioural & social sciences   [ ] Ecological, evolutionary & environmental sciences

For a reference copy of the document with all sections, see nature.com/documents/nr-reporting-summary-flat.pdf

Life sciences study design

All studies must disclose on these points even when the disclosure is negative.

Sample size	Sample sizes are indicated in the figure legends. For the serological analysis of Mpox convalescent individuals, sample size was limited by the number of available donors from a pre-existing cohort described in Yefet et al., iScience 26(2):105957. For antigen-specific B cell sorting, which formed the basis of monoclonal antibody generation, samples from seven Mpox convalescent donors were used, determined by the availability of samples within this cohort. The sample size for in vivo experiments was selected based on prior experience with similar infection models to ensure reproducibility and statistical robustness. No formal statistical power calculation was performed; sample sizes were considered sufficient to detect biologically meaningful differences based on previous studies.
Data exclusions	In one experiment involving C57BL/6 mice, a single animal died following blood collection before reaching the experimental endpoint. Consequently, its data were excluded from analyses at subsequent time points.
Replication	Replicates are indicated in the Figure legends
Randomization	All animals were randomly allocated into experimental groups.
Blinding	Blinding was not relevant to this study, as experimental conditions (e.g., recombinant protein expression, in vitro assays, and structural analyses) did not involve subjective assessments or group-based interventions. In the in vivo experiments, blinding was not possible due to the limited number of personnel authorized to work with infected animals.

Reporting for specific materials, systems and methods

We require information from authors about some types of materials, experimental systems and methods used in many studies. Here, indicate whether each material, system or method listed is relevant to your study. If you are not sure if a list item applies to your research, read the appropriate section before selecting a response.

Materials & experimental systems

n/a	Involved in the study	
☐	☒	Antibodies
☐	☒	Eukaryotic cell lines
☒	☐	Palaeontology and archaeology
☐	☒	Animals and other organisms
☒	☐	Clinical data
☒	☐	Dual use research of concern
☒	☐	Plants

Methods

n/a	Involved in the study	
☒	☐	ChIP-seq
☐	☒	Flow cytometry
☒	☐	MRI-based neuroimaging

Antibodies

Antibodies used

All anti-A35, H3 and A28 mAbs were produced in our lab from the BCR sequence originating from isolated B cells.
DNA of MGO53 was provided by the lab of Prof. Michel Nussenzweig, Rockefeller University and produced in our lab.
The DNA sequence of mAb 7D11 was obtained from the PDB structure 2I9L and produced in our lab as human IgG1.
Anti-Fab VHH as described in June E, et al., Journal of molecular biology 430.3 (2018): 322-336.

Commercial antibodies:
Labeling check reagent-VioBlue Miltenyi Biotec Cat#130-116-659 (diluted 1:100)
Anti-IgG FITC Miltenyi Biotec Cat#130-118-340; RRID:AB_2733672 (diluted 1:100)
Anti-rabbit Alexa Fluor 488 Invitrogen Cat#A11008 ; RRID:AB_143165 (diluted 1:400)
Anti-human IgG HRP Jackson ImmunoResearch Labs Cat#109-035-088; RRID:AB_2337584 (diluted 1:5000)
Anti-mouse IgG HRP Jackson ImmunoResearch Labs Cat#115-035-062 (diluted 1:5000)
Anti-rabbit HRP Jackson ImmunoResearch Labs Cat#111-035-144 (diluted 1:5000)
Anti-Avi-tag Avidity Cat#AbC (diluted 1:5000)
Anti-m13 bacteriophage provided by Prof. Jonathan Gershoni (diluted 1:5000)
Vaccinia immune globulin Omrix Cat#Omr-IgG-am ™ 5% IV (serial dilution-specified in the figure legends)
Rabbit polyclonal anti-VACV antibody Invitrogen Cat#PA1-7258; RRID:AB_561894 (diluted 1:6000)
Anti-IgG-Alexa Fluor 647 BioLegend Cat#BLG-410714 (diluted 1:100)
Anti-C1q-Alexa Fluor 647 Santa Cruz Cat#sc-53544 AF647 (diluted 1:100)
Anti-C1q Santa Cruz Cat#sc-53544
Anti-C3 BioLegend BLG-846302
Anti-human IgG gold nanoparticles (10 nm) Abcam Cat#AB-ab39596
Anti-mouse gold nanoparticles (20 nm) Abcam Cat#AB-ab270557-1
Anti-B220-PerCP-Cy5.5 Invitrogen Cat#07131-70-100 (diluted 1:100)
Anti-GL7-Alexa Fluor 647 BioLegend Cat#BLG-144606 (diluted 1:100)
Anti-FAS(CD95)-PE-Cy7 Miltenyi Biotec Cat#130-120-291 (diluted 1:100)
Anti-CD3-APC-Cy7 BioLegend Cat#BLG-317342 (diluted 1:100)
Anti-CD4-BV421 BioLegend Cat#BLG-405327 (diluted 1:100)
Anti-CD8a-FITC BioLegend Cat#BLG-100706 (diluted 1:100)
Anti-IgG-BV605 BioLegend Cat#BLG-405327 (diluted 1:100)
Anti-IgM-FITC BioLegend Cat#BLG-406506 (diluted 1:100)

Validation

All anti-A35, H3 and A28 mAbs were validated for their binding against their respected target antigen as shown in figure 1e and their sequence as descibed in the Supplemental Dataset.
MGO53 was validated in Wardemann H et al, Science 2003.
7D11 is an anti-M1 (VACV L1) mAb was previously described and its structure solved in Su. et al., Virology, 2007
The validations of commercially available antibodies used were in the technical data sheets provided by the manufacturers on their website.

Eukaryotic cell lines

Policy information about cell lines and Sex and Gender in Research

Cell line source(s)

Expi293F cells (Thermo Fisher Scientific, CAT#A14527)
HeLa (human female cervical cancer, ATCC CCL-2)
Vero (African green monkey kidney epithelial cells, ATCC CCL-81)
U2OS (human female osteosarcoma cells, ATCC HTB-96)

Authentication	The cell lines are regularly used and checked for growth rate and morphology under a microscope. Cell stocks are replaced after 20 passages.
Mycoplasma contamination	Cell lines are measured routinely for Mycoplasma contamination and tested negative.
Commonly misidentified lines (See ICLAC register)	None of the cell lines used in this study are listed as misidentified in the International Cell Line Authentication Committee (ICLAC) database.

Animals and other research organisms

Policy information about studies involving animals; ARRIVE guidelines recommended for reporting animal research, and Sex and Gender in Research

Laboratory animals	Female C57BL/6JOlaHsd and BALB/cOlaHsd mice were purchased at 7 weeks of age from Harlan Laboratories.
Wild animals	N/A
Reporting on sex	All in vivo Vaccinia virus experiments were performed in female C57BL/6JOlaHsd and BALB/cOlaHsd mice. The choice of female mice was based on consistency with prior immunological experiments in our laboratory and because Vaccinia virus challenge studies are routinely performed in female mice in this field, enabling direct comparison with published work. Using female mice also reduces variability associated with aggression and dominance behaviors in group-housed males. Since only female mice were used, sex-based comparative analyses were not possible.
Field-collected samples	N/A
Ethics oversight	Experimentation complied with Tel Aviv University- Institutional Animal Care and Use Committee (permit number TAU - MD - IL - 2407 - 149 – 4, TAU - MD - IL - 2411 - 181 – 5 and TAU - MD - IL - 2502 - 104 - 5).

Note that full information on the approval of the study protocol must also be provided in the manuscript.

Plants

Seed stocks	N/A
Novel plant genotypes	N/A
Authentication	N/A

Flow Cytometry

Plots

Confirm that:

- [x] The axis labels state the marker and fluorochrome used (e.g. CD4-FITC).
- [ ] The axis scales are clearly visible. Include numbers along axes only for bottom left plot of group (a 'group' is an analysis of identical markers).
- [x] All plots are contour plots with outliers or pseudocolor plots.
- [x] A numerical value for number of cells or percentage (with statistics) is provided.

Methodology

Sample preparation	All sample methodologies are indicated in the Method section in the manuscript.
Instrument	Cytoflex 4L- Beckman Coulter and AriaIII- Beckman Coulter.
Software	Data collection was performed using Cytexpert software and analyzed using FlowJo.
Cell population abundance	Mpox antigen binding IgG+ B cells were sorted into individual well and immediately lysed. No post sort fractions were used for further analysis.
Gating strategy	All gating strategies are indicated in the relevant figure legend sections in the manuscript.

- [x] Tick this box to confirm that a figure exemplifying the gating strategy is provided in the Supplementary Information.
